# Supplementary material for: Reverse genetics and comparative pathogenesis of Lone Star virus
Source: J Virol. 2026 Sep 4;100(10):e01009-26. doi: 10.1128/jvi.01009-26 (PMC13712813; doi:10.1128/jvi.01009-26)
Supplement: Supplementary file 1 [file jvi.01009-26-s0001.pdf]

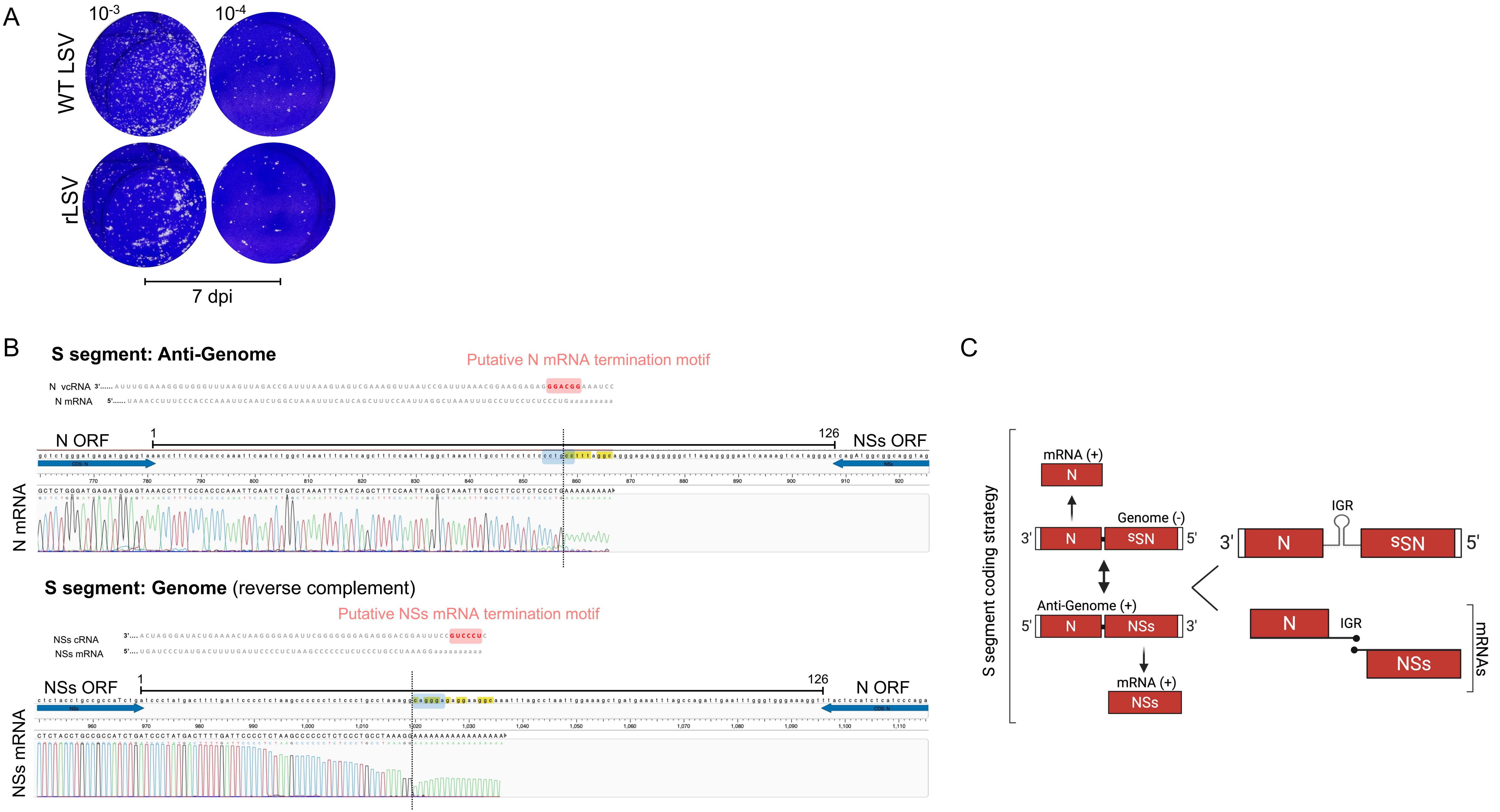

A
10-3
10-4
WT LSV
rLSV
7 dpi
B
S segment: Anti-Genome
Putative N mRNA termination motif
N vcRNA
N mRNA
N ORF
1
126
NSs ORF
N mRNA
S segment: Genome (reverse complement)
Putative NSs mRNA termination motif
NSs cRNA
NSs mRNA
NSs ORF
1
126
N ORF
NSs mRNA
C
S segment coding strategy
mRNA (+)
N
Genome (-)
3'
N
SSN
5'
Anti-Genome (+)
5'
N
NSs
3'
mRNA (+)
NSs
IGR
3'
N
SSN
5'
N
IGR
NSs
mRNAs
