# Supplementary material for: Reverse genetics and comparative pathogenesis of Lone Star virus
Source: J Virol. 2026 Sep 4;100(10):e01009-26. doi: 10.1128/jvi.01009-26 (PMC13712813; doi:10.1128/jvi.01009-26)
Supplement: Supplementary file 2 [file jvi.01009-26-s0002.pdf]

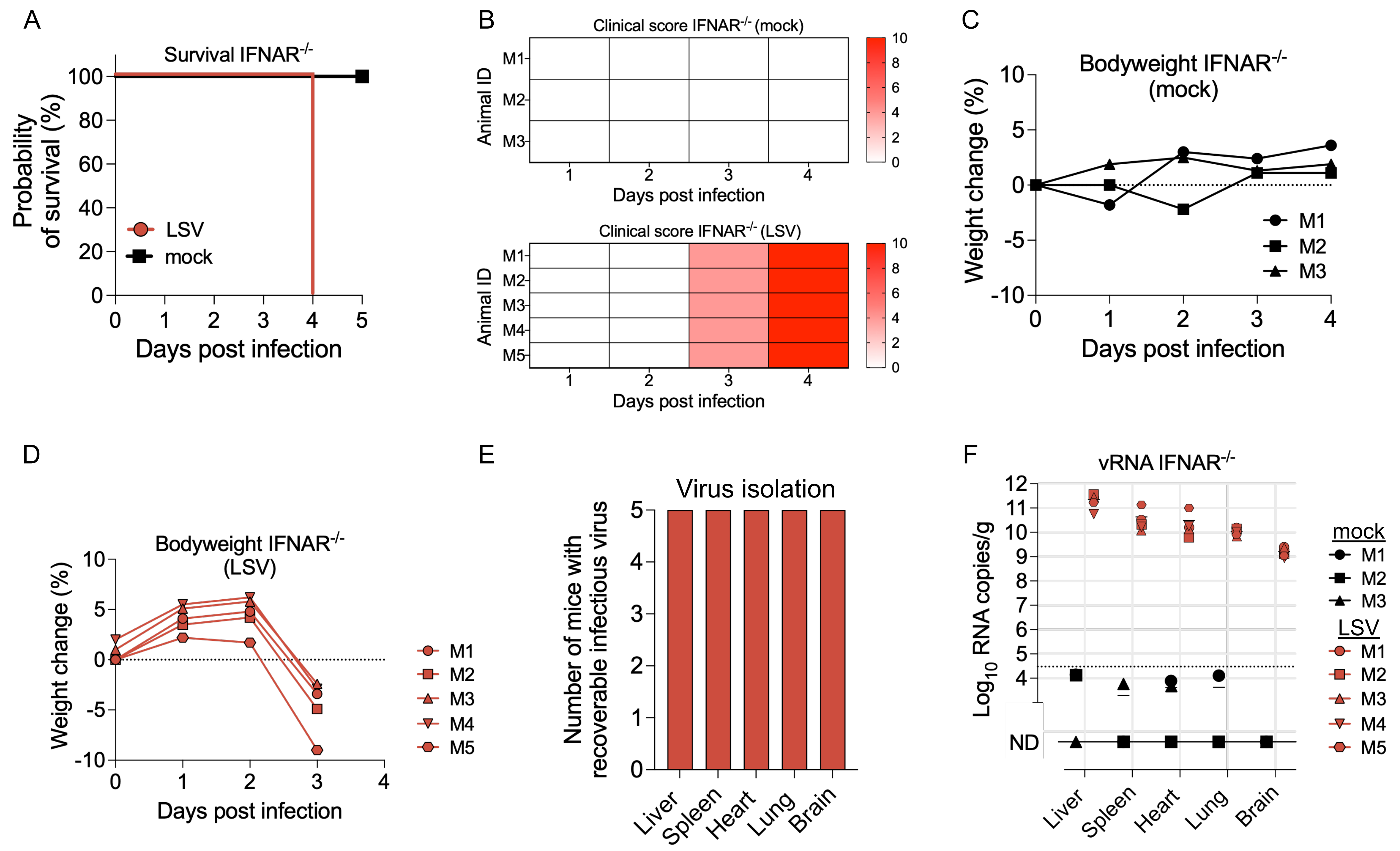

A
Survival IFNAR-/-
Probability of survival (%)
LSV
mock
Days post infection
B
Clinical score IFNAR-/- (mock)
Animal ID
M1
M2
M3
Days post infection
Clinical score IFNAR-/- (LSV)
M4
M5
C
Bodyweight IFNAR-/- (mock)
Weight change (%)
Days post infection
D
Bodyweight IFNAR-/- (LSV)
Weight change (%)
Days post infection
E
Virus isolation
Number of mice with recoverable infectious virus
Liver
Spleen
Heart
Lung
Brain
F
vRNA IFNAR-/-
Log10 RNA copies/g
ND
mock
LSV
