# Supplementary material for: Reverse genetics and comparative pathogenesis of Lone Star virus
Source: J Virol. 2026 Sep 4;100(10):e01009-26. doi: 10.1128/jvi.01009-26 (PMC13712813; doi:10.1128/jvi.01009-26)
Supplement: Supplementary file 3 [file jvi.01009-26-s0003.pdf]

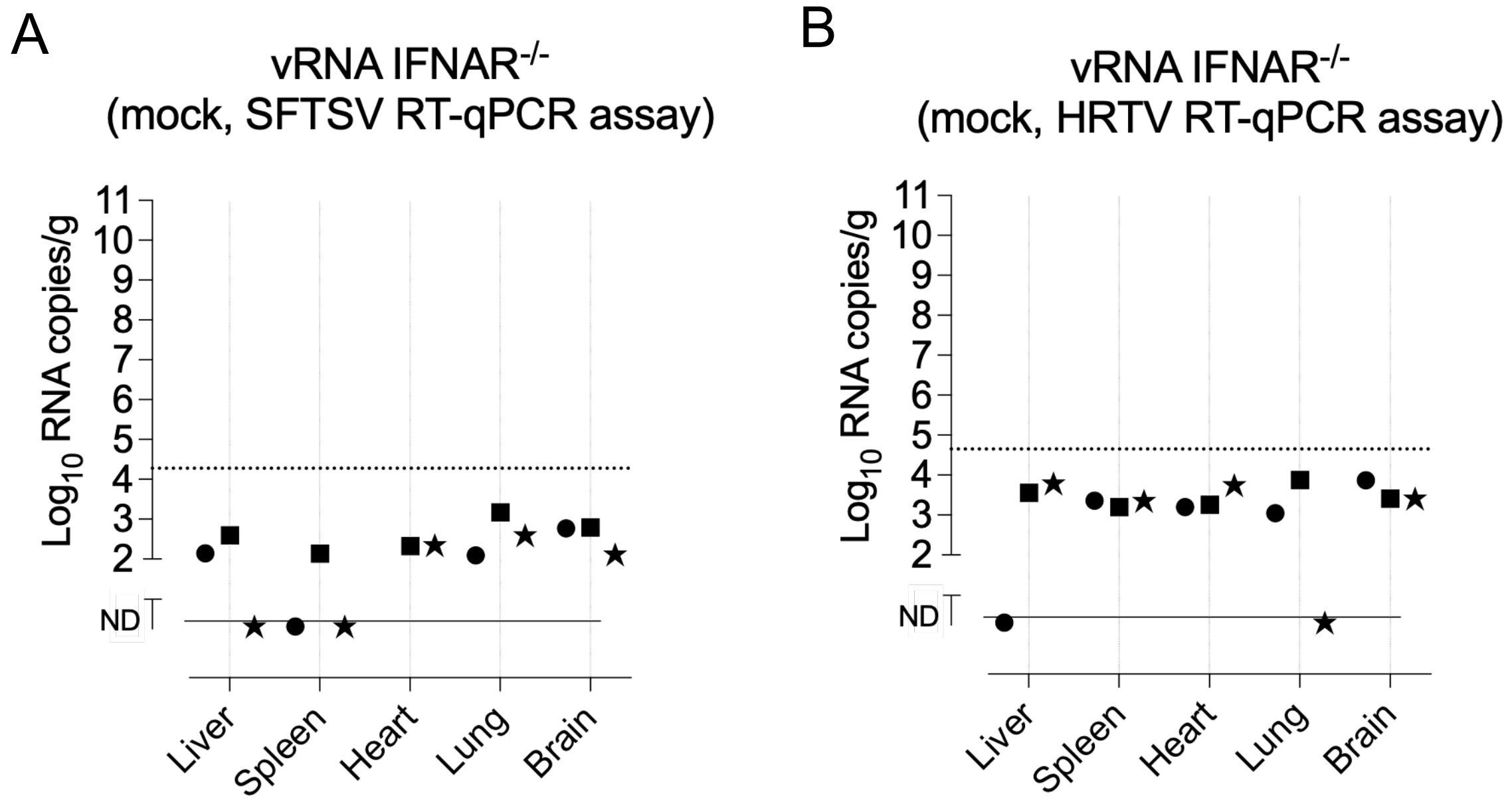
A
vRNA IFNAR-/-
(mock, SFTSV RT-qPCR assay)
Log10 RNA copies/g
11
10
9
8
7
6
5
4
3
2
ND
Liver
Spleen
Heart
Lung
Brain
B
vRNA IFNAR-/-
(mock, HRTV RT-qPCR assay)
Log10 RNA copies/g
11
10
9
8
7
6
5
4
3
2
ND
Liver
Spleen
Heart
Lung
Brain
