# Supplementary material for: Reverse genetics and comparative pathogenesis of Lone Star virus
Source: J Virol. 2026 Sep 4;100(10):e01009-26. doi: 10.1128/jvi.01009-26 (PMC13712813; doi:10.1128/jvi.01009-26)
Supplement: Supplementary file 4 [file jvi.01009-26-s0004.docx]

**Supplemental Figure 1. Plaque morphology and S-segment transcription termination mapping for LSV.** (A) Representative plaque morphology of WT LSV and rLSV on Vero E6 cells at the indicated dilutions. (B) Mapping of putative transcription termination motifs within the LSV S-segment IGR. The S-segment antigenome and genome orientations are shown with the N and NSs ORFs indicated. Representative Sanger sequencing traces of N and NSs mRNAs are shown beneath each map. Dotted vertical lines indicate the predicted 3′ ends of the N and NSs mRNAs, and highlighted sequences indicate nearby putative transcription termination motifs within the IGR. (C) Schematic representation of the LSV S-segment ambisense coding strategy and the relative positions of the N and NSs mRNAs, illustrating that both mapped mRNA 3′ ends are located within the IGR.

**Supplemental Figure 2. WT LSV causes rapid systemic disease in IFNAR^-/-^ mice.** IFNAR^-/-^ mice were infected SC with 10^6^ TCID_50_ WT LSV or mock-infected with Opti-MEM. (A) Survival curve of WT LSV-infected and mock-infected mice. (B) Clinical scores of mock-infected and WT LSV-infected mice. Clinical disease severity is depicted by increasing intensity of red. (C, D) Percent body weight change in mock-infected (C) and WT LSV-infected (D) mice. (E) Number of mice from which infectious virus was recovered from liver, spleen, heart, lung, and brain at necropsy. (F) vRNA loads in liver, spleen, heart, lung, and brain tissues from WT LSV-infected and mock-infected mice, measured by RT-qPCR and shown as log_10_ RNA copies/g tissue. The dashed line indicates the limit of detection. ND, not detected. Each symbol represents an individual mouse.

**Supplemental Figure 3. Mock-infected control tissues analyzed using virus-specific RT-qPCR assays.** (A) vRNA levels in mock-infected IFNAR^-/-^ mouse tissues were analyzed using the SFTSV-specific RT-qPCR assay. (B) vRNA levels in mock-infected IFNAR^-/-^ mouse tissues were analyzed using the HRTV-specific RT-qPCR assay. Tissue vRNA loads were measured by RT-qPCR and are shown as log_10_ RNA copies/g tissue. Dashed lines indicate the limit of detection. ND, not detected. Each symbol represents an individual mouse.

**Supplementary Table 1. Oligonucleotides used in this study.** Primer and probe sequences used for LSV genome sequencing, S-segment RACE, and virus-specific RT-qPCR assays for LSV, HRTV, and SFTSV. Primer/probe names, target virus, genome segment, sequence, and intended purpose are indicated.
