# Supplementary material for: Reverse genetics and comparative pathogenesis of Lone Star virus
Source: J Virol. 2026 Sep 4;100(10):e01009-26. doi: 10.1128/jvi.01009-26 (PMC13712813; doi:10.1128/jvi.01009-26)
Supplement: Supplementary file 5 [file jvi.01009-26-s0005.docx]

**Supplementary Table 1:** Oligonucleotides used in this study

| **Virus** | **Segment** | **Primer name** | **Sequence (5' - 3')** | **Purpose** |
| --- | --- | --- | --- | --- |
| LSV | S | priT7LSV-S-F | CGAAATTAATACGACTCACTATAGACACAAAGAGGCCGCTAATAG | sequencing |
|  |  | priT7LSV-S-R | GGAGGTGGAGATGCCATGCCGACCCACACAGAGAGGCCGCACCATG |  |
|  | M (fragment 1) | priT7LSV-M-F | GAAATTAATACGACTCACTATAGACACAAAGACGCCAGCTAC |  |
|  |  | priT7LSV-M1-R | GAGAATGCAACCCACTAGCCCC |  |
|  | M (fragment 2) | priT7LSV-M2-F | CAGCTCTGGTTGCTCCGGAGTTG |  |
|  |  | priT7LSV-M-R | CGAGGAGGTGGAGATGCCATGCCGACCCACACAAAGACCGCCAGCATTC |  |
|  | L (fragment 1) | priT7LSV-L-F | CGAAATTAATACGACTCACTATAGACACAAAGACGCCAACATG |  |
|  |  | priT7LSV-L1-R | GGGCAGTAGAGGTTCTGTCACAAACC |  |
|  | L (fragment 2) | priT7LSV-L2-F | GGCCTTAACTGAGGAATTGATTACC |  |
|  |  | priT7LSV-L2-R | CATACCTGTTCGGTTCTTAATACCG |  |
|  | L (fragment 3) | priT7LSV-L3-F | GGAGGGTCTCTCTCCAAATGCCATTTGATCAG |  |
|  |  | priT7LSV-L-R | GAGGAGGTGGAGATGCCATGCCGACCCACACAAAGACCGCCAACACC |  |
| LSV | S | Adaptor | GACTCGAGTCGACATCG | RACE |
|  | S | Adaptor-dT17 | GACTCGAGTCGACATCGATTTTTTTTTTTTTTTTTT |  |
|  | S | priLSV-NP-GSP | GAGATGGAGTAAACCTTTCCCAC |  |
|  | S | priLSV-NSs-GSP | CATAGGGATCAGATGGCGGCAG |  |
| LSV | S | rLSV-S-qPCRprimers (Fwd) | GTGGGAAAGGTTTACTCCATCT | RT-qPCR |
|  |  | rLSV-S-qPCRprimers (Rev) | CTTCAGGGTCTTGGGATCTTG |  |
|  |  | rLSV-S-qPCRprobe (FAM) | ACCAGAAGAAGAAAGCGGTGGAGT |  |
| HRTV | S | HRTV-R99-SqPCRprimers (Fwd) | GCTTACATGTTTCTGTAAGCAGCAG | RT-qPCR |
|  |  | HRTV-R99-SqPCRprimers (Rev) | CTTCTTCCCAAATGCCACCAG |  |
|  |  | HRTV-R99-SqPCRprobe (FAM) | ATCAGCTGGCTGCAGGCCAAG |  |
| SFTSV | S | SFTSV-CB8-SqPCRprimers (Fwd) | TGGGAAAGAAGACAGAGTTCAC | RT-qPCR |
|  |  | SFTSV-CB8-SqPCRprimers (Rev) | GATGCCTTCACCAAGACTATCA |  |
|  |  | SFTSV-CB8-SqPCRprobe (FAM) | CGGAGCCAGCAAGACAGAAGTTTACA |  |
